# Fluorescence perfusion assessment of vascular ligation during ileal pouch-anal anastomosis

**DOI:** 10.1007/s10151-022-02666-1

**Published:** 2022-09-21

**Authors:** M. D. Slooter, E. M. L. van der Does de Willebois, J. J. Joosten, M. A. Reijntjes, C. J. Buskens, P. J. Tanis, W. A. Bemelman, R. Hompes

**Affiliations:** Department of Surgery, Amsterdam UMC, University of Amsterdam, Cancer Centre Amsterdam, Academic Medical Centre (AMC), Meibergdreef 9, Postbox 22660, 1100 DD Amsterdam, The Netherlands

**Keywords:** Fluorescence angiography, Indocyanine green (ICG), Ileal pouch-anal anastomosis (IPAA), Vascular ligation, Anastomotic leakage

## Abstract

**Background:**

Intraoperative fluorescence angiography (FA) is of potential added value during ileal pouch-anal anastomosis (IPAA), especially after vascular ligation as part of lengthening measures. In this study, time to fluorescent enhancement during FA was evaluated in patients with or without vascular ligation during IPAA.

**Methods:**

This is a retrospective cohort study of all consecutive patients that underwent FA-guided IPAA between August 2018 and December 2019 in our tertiary referral centre. Vascular ligation was defined as disruption of the ileocolic arcade or ligation of interconnecting terminal ileal branches. FA was performed before and after ileoanal anastomotic reconstruction. During FA, time to fluorescent enhancement was recorded at different sites of the pouch.

**Results:**

Thirty-eight patients [55.3% male, median age 45 years (IQR 24–51 years)] were included, of whom the majority (89.5%) underwent a modified-2-stage restorative proctocolectomy. Vascular ligation was performed in 15 patients (39.5%), and concerned central ligation of the ileocolic arcade in 3 cases, interconnecting branches in 10, and a combination in 2. For the entire cohort, time between indocyanine green (ICG) injection and first fluorescent signal in the pouch was 20 s (IQR 15–31 s) before and 25 s (IQR 20–36 s) after anal anastomotic reconstruction. Time from ICG injection to the first fluorescent signal at the inlet, anvil and blind loop of the pouch were non-significantly prolonged in patients that received vascular ligation.

**Conclusions:**

Results from this study indicate that time to fluorescence enhancement during FA might be prolonged due to arterial rerouting through the arcade or venous outflow obstruction in case of vascular ligation.

## Introduction

Ileal pouch-anal anastomosis (IPAA) is a surgical procedure to restore continuity after proctocolectomy for inflammatory bowel disease or inherited colorectal cancer disorders. After IPAA, anastomotic leakage is a severe complication occurring in up to 15% of patients [1–3]. Patients with anastomotic leakage are at risk of developing pouch dysfunction or failure due to chronic pelvic sepsis, which can only be solved by complex salvage surgery or pouch excision.

**Fig. 1:**
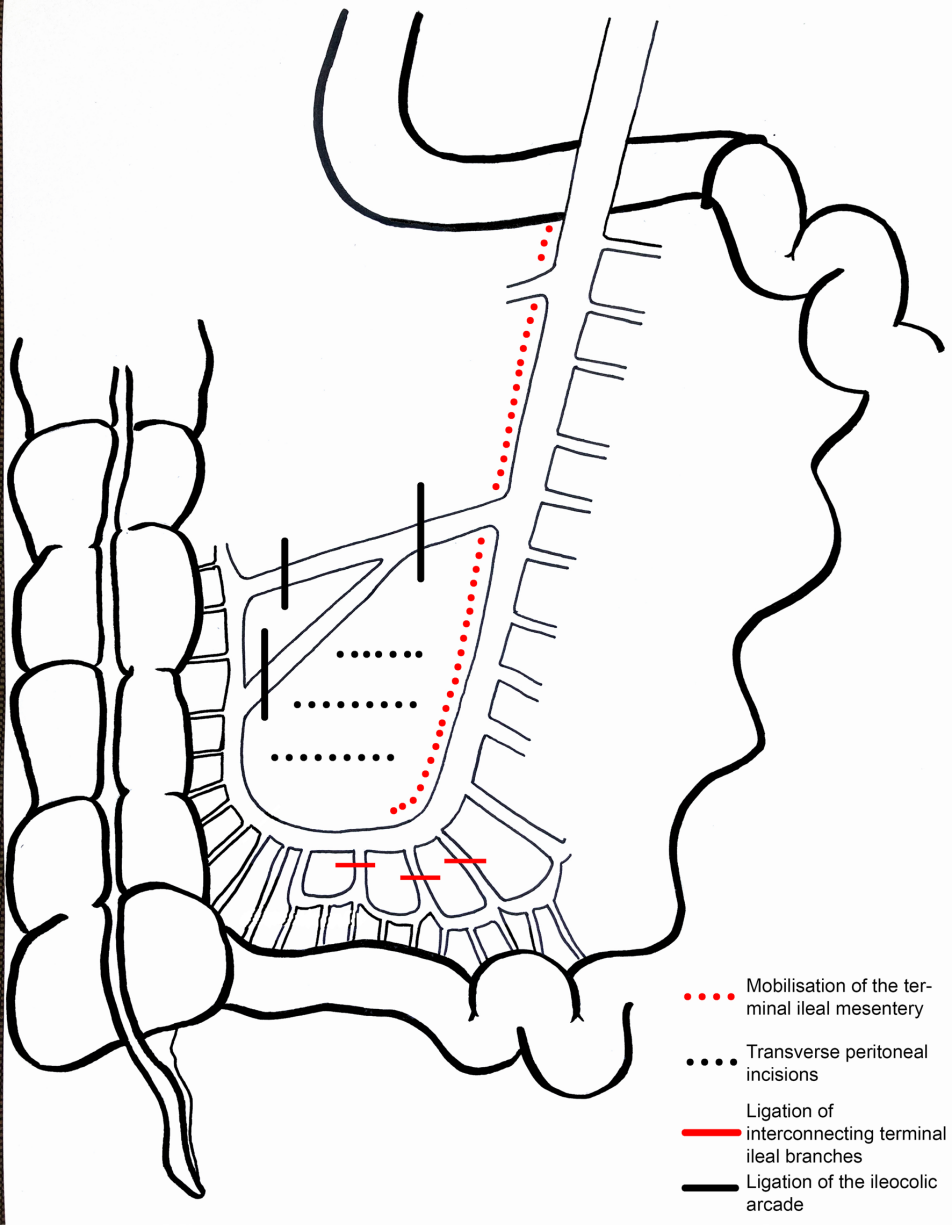
Lengthening manoeuvres of the ileal pouch

Essential in the prevention of anastomotic leaks is a tension-free and well-vascularized ileoanal anastomosis. However, balancing between tension and perfusion is challenging as these two factors may oppose one another. Additional length of the terminal ileal mesentery can be achieved by mobilisation of the mesenteric root to the level of the duodenum and transverse peritoneal incisions. When sufficient length cannot be achieved by these basic lengthening manoeuvres, vascular ligations might be required. The ileocolic arcade or interconnecting terminal ileal branches can be sacrificed to gain extra length [4] (Fig. 1). Hereby the pouch can be exposed to vascularisation problems, by disruption of in- or outflow [5].

Intraoperative fluorescence angiography (FA) using indocyanine green (ICG) is an emerging technique that is widely applied to assess bowel perfusion [6]. During IPAA, FA is proposed to be of added value, especially after vascular ligation [7–9]. However, its current interpretation is subjective, and does not routinely discriminate between an inflow or outflow problem. Time dependent change of FA appears more objective and is a promising method for quantification predicting both in- or outflow problems [10].

The primary objective of the present study was to assess the time dependent FA characteristics of ileoanal pouches with intact vascularisation as opposed to pouches where vascular lengthening measures are required.

## Materials and methods

This was a retrospective cohort study of all consecutive patients that underwent FA-guided IPAA in a tertiary referral centre, from introduction of FA in August 2018 until December 2019. Two groups were compared: patients with and without vascular ligation. Patients were excluded if they had undergone redo IPAA, when FA was not performed, or when FA was performed using another imaging platform than specified below. Since introduction of FA, FA data were routinely recorded in the electronic medical record. Patient data were retrospectively collected from the electronic medical records.

The Institutional Review Board of the Amsterdam University Medical Centres (UMC), location Academic Medical Centre (AMC), approved the study protocol and confirmed that the Medical Research involving Human Subjects Act (WMO) did not apply. In compliance to the General Data Protection Regulation, need for written informed consent was waived due to the retrospective nature of the study. All patients were sent information concerning the study including an opt-out letter. If patients did not reply within 4 weeks, approval for use of data was assumed.

### Fluorescence-guided IPAA

Different strategies for restorative proctocolectomy with IPAA were applied [11]. Completion proctectomy and IPAA were performed as previously described [12]. At our centre, the ileocolic pedicle management method at time of subtotal colectomy is to preserve the ileocolic arcade. The ileocolic artery can be ligated at different levels to make the pouch reach the pelvic floor if lengthening manoeuvres are required, as indicated in Fig. 1. Either a stapled or hand-sewn IPAA was created. A hand-sewn IPAA was preceded by mucosectomy of the rectal stump.

Perfusion of the J-pouch was assessed before (serosal assessment) and after (mucosal assessment) ileoanal anastomotic reconstruction. Serosal assessment was performed after J-pouch construction, however in a minority of cases this was done before pouch creation after final decision on vessel ligation. Visual inspection was followed by ICG injection (0.1 mg/kg/bolus) for FA. Imaging was performed by laparoscopic PINPOINT or hand-held Spy-phi (Stryker, Kalamazoo, MI, U.S.A.).

In case of anastomotic leaks, reoperation was performed for ileostomy creation, if not performed primarily. This was followed by immediate surgical transanal closure of the defect if small, Endo-SPONGE^®^ (B. Braun Surgical S.A., Barcelona, Spain) vacuum assisted closure (EVAC) of the defect [13], or restorative redo surgery at a later stage.

### Outcomes

**Fig. 2:**
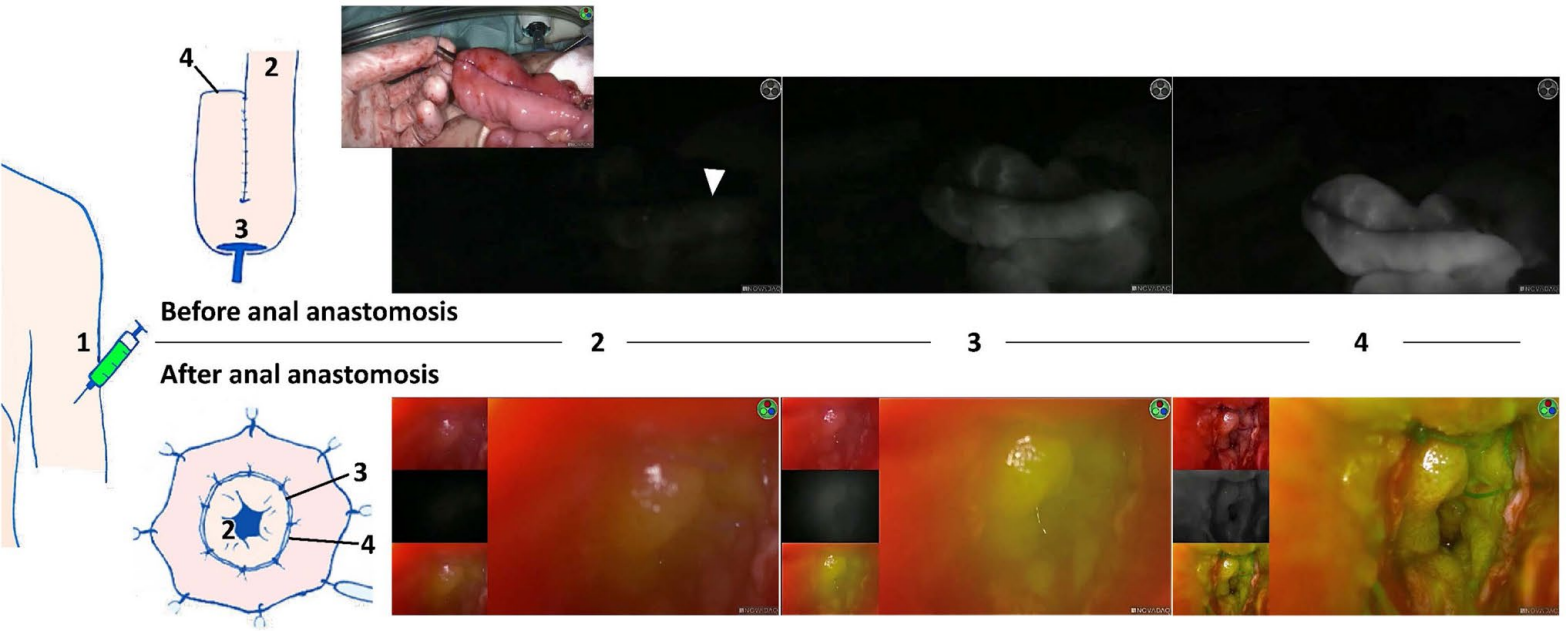
Time-points to assess time to fluorescent enhancement during ileal pouch-anal anastomosis. Before IPAA reconstruction, the J-pouch was examined and the following time-points were recorded during FA: 1. time of ICG injection (ICGi), 2. time of fluorescent signal at the inlet of the pouch (inlet), 3. at the planned anastomotic site (anvil), and 4. at the blind loop of the J-pouch (blind loop). After ileoanal anastomotic reconstruction, the anastomosis was evaluated transanally and FA time-points included: 1. time of ICG injection (ICGi), 2. time of first fluorescent signal of the mucosa (first signal), 3. time of fluorescence of the pouch at the anal anastomotic site (pouch-anal anastomosis), and 4. of the distal cuff at the anal anastomotic site (distal cuff-anal anastomosis)

The primary outcome was time to fluorescent enhancement, a quantitative FA characteristic, in patients with or without vascular ligation. Details on vascular ligation were retrieved from the surgical report and was scored when the ileocolic arcade or interconnecting terminal ileal branches (referred to as: interconnecting branches) were ligated. Assessment of time to fluorescent enhancement is displayed in Fig. 2. Time-points were manually assessed using a digital clock and recorded. If sites changed due to change in management, time-points were adjusted accordingly. Time values were the difference between time-points in seconds, with time-point of ICG injection, inlet/first signal, or the anvil/pouch-anal anastomosis as *t* = 0.

Secondary outcomes included other FA characteristics, including change in management and additional surgical time, haemodynamic parameters during FA, anastomotic leakage within 90 days, postoperative stay and mortality rate within 90 days. The interpretation of the FA was at surgeon’s discretion. Change in management was defined as every measure taken based on the results of FA only. Change of management was mainly based on small versus large areas of poor or no fluorescent signal versus delayed fluorescent enhancement and to a lesser extent the time to fluorescence. Change in management included suture reinforcement of hypoperfused regions, additional resections, selecting a more proximal loop for pouch creation or ileostomy creation. Additional surgical time was recorded from beginning to end of the fluorescent mode in minutes. Haemodynamic parameters included mean arterial pressure, heart rate and noradrenaline usage and dosage. Anastomotic leakage was recorded when an anastomotic defect was objectified by computed tomography (CT)-scan, during endoscopy or reoperation, and was graded according to impact on clinical management [1]. For anastomotic leakage, details were collected including location, signs of ischaemia and retraction, type of management and time to stoma closure.

### Statistical analysis

All categorical data were presented as number of cases and percentages, whilst continuous data were shown as either mean ± standard deviation or as median and interquartile range (IQR) or total range, depending on data distribution. Categorical variables were compared using a *χ*^2^ test or Fisher’s exact test. Comparison of continuous variables was done with a t-test or Mann–Whitney *U* test, according to distribution. A *p* value ≤ 0.05 was considered statistically significant. Time values were reported as median and IQR and were primarily assessed for patients with or without vascular ligation, and secondary for patients with or without change in management or anastomotic leakage. Time values including ‘distal cuff-anal anastomosis’ were not included in the comparison for vascular ligation. Time values were compared between groups using the Mann–Whitney *U* test. A *p* value ≤ 0.05 was considered statistically significant. Univariate logistic regression analysis was performed to evaluate predictive time values for anastomotic leakage. When analysis revealed a *p* value below 0.2 [14], a receiver operating characteristic (ROC) curve was produced. When the ROC-curve generated an area under de curve (AUC) above 0.7, a cut-off value was produced with high specificity using Youden’s statistics. Correlations between time values and haemodynamic parameters were assessed by calculating the Spearman’s rank correlation coefficient *ρ*. A *p* value ≤ 0.05 was considered statistically significant.

Data were analysed using the Statistical Package for Social Sciences (SPSS) (IBM Statistics, IBM Corp., Armonk, NY, USA), version 26.0.

## Results

In total 42 patients underwent IPAA during the study period, 4 of whom were excluded because of redo IPAA (*n* = 1), non FA-guided IPAA (*n* = 2) or another FA imaging platform (*n* = 1). Thus, 38 patients were included, [55.3% male, median age 45 years (IQR 24–51 years)] (Table 1). The majority of patients (89.5%) underwent IPAA for ulcerative colitis (UC). Other diagnosis included Crohn’s disease (*n* = 1), familial adenomatous polyposis (*n* = 1), Lynch syndrome (*n* = 1), and Lynch syndrome in combination with MUTYH-associated polyposis (*n* = 1). Comorbidities are shown in Table 1. The majority of patients (89.5%) underwent a modified-2-stage (Table 1). Surgical details are shown in Table 1.

**Table 1:** Baseline characteristics and operative details

	FA-guided IPAA (*N* = 38)	Vascular ligation (*n* = 15)	No vascular ligation (*n* = 23)
Male sex	21 (55.3)	11 (73.3)	10 (43.5)
Age (years) median IQR	45 (24–51)	42 (22–58)	45 (25–51)
BMI (kg/m^2^) median IQR	24.6 (21.5–27.6)	23.5 (21.5–26.9)	24.7 (21.4–29.3)
ASA classification (≤ 2)	38 (100)	15 (100)	23 (100)
Smoker (active)	6 (15.8)	4 (26.7)	2 (8.7)
Comorbidity^a^	5 (13.2)	2 (13.3)	3 (13.0)
Cardiovascular	0 (0)	0 (0)	0 (0)
Pulmonary	4 (10.5)	2 (13.3)	2 (8.7)
Diabetes	1 (2.6)	0 (0)	1 (4.3)
Diagnosis			
Ulcerative colitis	34 (89.5)	12 (80.0)	22 (95.7)
Other	4 (10.5)	3 (20.0)	1 (4.3)
Stage procedure			
1-stage	1 (2.6)	1 (6.7)	0 (0)
2-stage	1 (2.6)	1 (6.7)	0 (0)
Modified 2-stage	34 (89.5)	11 (73.3)	23 (100)
Other	2 (5.3)	2 (13.3)	0 (0)
Immunomodulating medication^b^	4 (10.5)	2 (13.3)	2 (8.7)
Abdominal approach			
Laparoscopy^c^	32 (84.2)	15 (100)	17 (73.9)
Open	6 (15.8)	0 (0)	6 (21.6)
Conversion	2 (5.3)	2 (13.3)	0 (0)
Construction anastomosis			
Hand-sewn	1 (2.6)	1 (6.7)	0 (0)
Stapled	37 (97.4)	14 (93.3)	23 (100)
Additional lengthening manoeuvres			
Transverse peritoneal incisions	31 (81.6)	15 (100)	16 (69.6)
Vascular ligation	15 (39.5)	15 (100)	0 (0)
Vascular ligation specification			
Ileocolic arcade	3 (7.9)	3 (20.0)	–
Interconnecting branches	10 (26.3)	10 (66.7)	–
Combination of ileocolic arcade and interconnecting branches	2 (5.3)	2 (13.3)	–
Intraoperative complications^d^	1 (2.6)	1 (6.7)	0 (0)

Data is shown in *n* (%), unless stated otherwise

*ASA* American Society of Anesthesiologists classification, *AMC* Amsterdam University Medical Centres location Academic Medical Centre, *BMI* body-mass index, *FA* Fluorescence angiography, *IPAA* Ileal pouch-anal anastomosis, *IQR* Interquartile range

^a^Vascular comorbidity: brain infarction, myocardial infarction, or peripheral vascular disease. Pulmonary comorbidity: asthma or COPD. Diabetes: type 1 or 2

^b^Mesalazine, or biological within 3 months before surgery

^c^Includes single/multi-port and hand-assisted

^d^Complications: bleeding (> 500 cc)

### Primary outcome

During IPAA, vascular ligation was performed in 15 of 38 patients (39.5%). In 3 of these cases, the ileocolic arcade was ligated, in 10 interconnecting branches, and in 2 a combination. FA was performed before and after ileoanal anastomosis in 29 patients (76.3%), in 6 patients (15.8%) only before anastomosis and in 3 patients (7.9%) only after. Time values for the entire cohort and comparison for vascular ligation are shown in Table 2. Time values before anastomosis with ICGi as *t* = 0 were prolonged, although not significantly, in patients with vascular ligation. No differences were observed for time values once ICG was observed in the pouch (times with inlet or anvil as *t* = 0). After anastomosis, no time differences were observed, except for a longer (non-significant) time interval between ICG injection and first signal.

**Table 2:** Time to fluorescent enhancement: time values for patients with or without vascular ligation

	*t* = 0	End	Overall (*n* = 38)	Vascular ligation (*n* = 15)	No vascular ligation (*n* = 23)	*p* value
			Time to fluorescence (sec)			
Before anastomosis^a^	ICG injection	Inlet	20 (15–31)	24 (17–34)	19 (14–27)	0.294
		Anvil	28 (20–34)	31 (26–36)	26 (19–31)	0.253
		Blind loop	31 (23–52)	35 (28–52)	25 (21–50)	0.150
	Inlet	Anvil	3 (2–6)	3 (2–5)	4 (2–7)	0.393
		Blind loop	6 (4–14)	6 (2–20)	5 (4–13)	0.961
	Anvil	Blind loop	2 (0–4)	2 (0–6)	2 (0–5)	0.584
After anastomosis^b^	ICG injection	First signal	25 (20–36)	28 (25–34)	22 (19–38)	0.345
		Pouch-anal anastomosis	40 (24–45)	40 (30–40)	40 (23–51)	0.797
	First signal	Pouch-anal anastomosis	4 (2–10)	4 (2–12)	3 (1–19)	0.913

Data is shown as median and interquartile range

*ICG* indocyanine green

^a^Measurements for 33/35 cases: 15 patients with and 18 patients without vascular ligation

^b^Measurements for 29/32 cases: 9 patients with and 20 patients without vascular ligation

### Secondary outcomes

FA findings led to change in management in 7 patients (18.4%) (Table 3), due to absence of ICG fluorescence in parts of the pouch in 6 cases and to delayed ICG fluorescence in one. Absence of ICG fluorescence was noticed in a small area of the pouch (< 1 cm of bowel) in 4 patients. In 2 patients, the non-fluorescent part was at the blind loop after pouch construction and was additionally resected. In the 2 other patients, the non-fluorescent area was in close proximity to the anvil or at the longitudinal stapler line, and was reinforced by sutures. In 2 patients, absence of fluorescence in a segment of terminal ileum (10 cm and 25 cm) led to selection of a more proximal loop for pouch creation. Intact but delayed ICG fluorescence was noticed in 1 patient between anvil and blind loop (33 s) and an ileostomy was created.

**Table 3:** Change in management due to fluorescence angiography in patients with or without vascular ligation

	Vascular ligation (*N* = 15)	No vascular ligation (*N* = 23)
No change in management	9 (60.0)	22 (95.7)
Change in management	6 (40.0)	1 (4.3)
Suture reinforcement^a^	2/6 (33.3)	0/1 (0)
Additional bowel resection	2/6 (33.3)	0/1 (0)
Selection of a more proximal loop	1/6 (16.7)	1/1 (100)
Creation of ileostomy	1/6 (16.7)	0/1 (0)

Data is shown in *n* (%) or *n*/*n* (%), unless otherwise stated

^a^Excludes routinely performed anastomotic reinforcement

Change in management occurred more often after vascular ligation (6/15 (40.0%) vs 1/23 (4.3%); *p* = 0.010) (Table 3). In case of ligation of the ileocolic arcade, change in management occurred in one out of three cases in which additional resection of the blind loop was performed. In case of ligation of interconnecting branches, change in management occurred in four out of ten cases and included suture reinforcement (2/4), additional resection of the blind loop (1/4) or ileostomy creation (1/4). When ligation of both ileocolic arcade and interconnecting branches was performed, 1 out of 2 patients required pouch reconstruction using a more proximal loop. In the latter case, a new pouch was reconstructed after absence of fluorescence in the entire pouch subsequently to ligation of the ileocolic arcade. For adequate length of the new pouch, interconnecting branches were additionally ligated. However, in this case, no intact arcade was present and only segmental branches, leading to an additional resection of 25 cm of small bowel. The pouch was created with ICG-perfused small bowel and anastomosed under acceptable tension. No significant differences were found for time values between patients with or without change of management (data not shown).

Median additional operative time owing to FA was 3 min (IQR 2–3 min). Time values were not correlated to haemodynamic parameters (*p* > 0.05), except for heart rate that was inversely correlated to time value ICGi-inlet (*p* = 0.024).

Median postoperative hospital stay was 6 days (IQR 6–14 days). Anastomotic leakage was observed in 6 patients (15.8%). All anastomotic defects were located on the circular anastomosis without signs of ischaemia or retraction. In 5 of 6 cases of leakage, reoperation was required to create an ileostomy making it a Grade C leak. For management of anastomotic leakage, 4 patients underwent EVAC and 2 had immediate transanal closure of the anastomotic defect. Of the 5 patients that received a secondary ileostomy, 4 had stoma closure after a median of 177 days (total range 131–323 days) after IPAA, and 1 patient is still undergoing EVAC therapy.

With regard to the long -term results, 83% of patients with an anastomotic leakage (5/6) had a functional anastomosis after a median follow up of 28 months (IQR 24–33 months). The only patient with a stoma still in situ is a patient with UC and dysplasia, who had already undergone neoadjuvant chemoradiation and a low anterior resection complicated by a chronic leak, for which the patient underwent a resection of the leaking anastomosis with IPAA.

There was no mortality in this cohort. Occurrence of anastomotic leakage did not significantly differ between patients with or without vascular ligation (2/15 (13.3%) vs 4/23 (17.4%), respectively; *p* = 1.000). Two anastomotic leaks in patients with vascular ligation occurred after ligation of interconnecting branches, one of which was combined with ligation of the ileocolic arcade. Anastomotic leakage was observed in 2 of 7 patients with a change in management (28.6%), compared to 4 of 31 (12.9%) when the procedure remained unchanged (*p* = 0.302).

Comparison of time values in patients with or without anastomotic leakage are shown in Table 4. Time values including transit time through the pouch were non-significantly prolonged in patients with anastomotic leakage (anvil-blind loop, time values to pouch-anal anastomosis). Time from ICGi to pouch-anal anastomosis was predictive for anastomotic leakage (*p* = 0.135, AUC = 0.734). A cut-off value of 53 s was derived to predict anastomotic leakage (specificity 100%, sensitivity 50%, positive predictive value 100%, and negative predictive value 89%).

**Table 4:** Time to fluorescent enhancement: times values for patients with or without anastomotic leakage

	*t* = 0	End	Anastomotic leakage (*N* = 6)	No anastomotic leakage (*N* = 32)	*p* value
			Time to fluorescence (sec)		
Before anastomosis^a^	ICG injection	Inlet	20 (15–38)	20 (15–27)	0.763
		Anvil	30 (17–42)	28 (23–32)	0.970
		Blind loop	39 (23–50)	29 (23–52)	0.749
	Inlet	Anvil	3 (2–4)	3 (2–7)	0.693
		Blind loop	9 (3–20)	5 (4–14)	0.655
	Anvil	Blind loop	6 (1–16)	2 (0–3)	0.247
After anastomosis^b^	ICG injection	First signal	29 (11–44)	25 (20–35)	0.644
		Pouch-anal anastomosis	47 (31–77)	40 (23–40)	0.135
		Distal cuff-anal anastomosis	49 (37–78)	41 (32–51)	0.348
	First signal	Pouch-anal anastomosis	8 (2–40)	3 (2–12)	0.444
		Distal cuff-anal anastomosis	13 (5–41)	14 (5–25)	0.893
	Pouch-anal anastomosis	Distal cuff-anal anastomosis	2 (0–8)	4 (0–13)	0.470

Data is shown as median and interquartile range

*ICG* indocyanine green

^a^Measurements for 33/35 cases: 5 patients with and 28 patients without anastomotic leakage

^b^Measurements for 29/32 cases: 5 patients with and 24 patients without anastomotic leakage

**Fig. 3:**
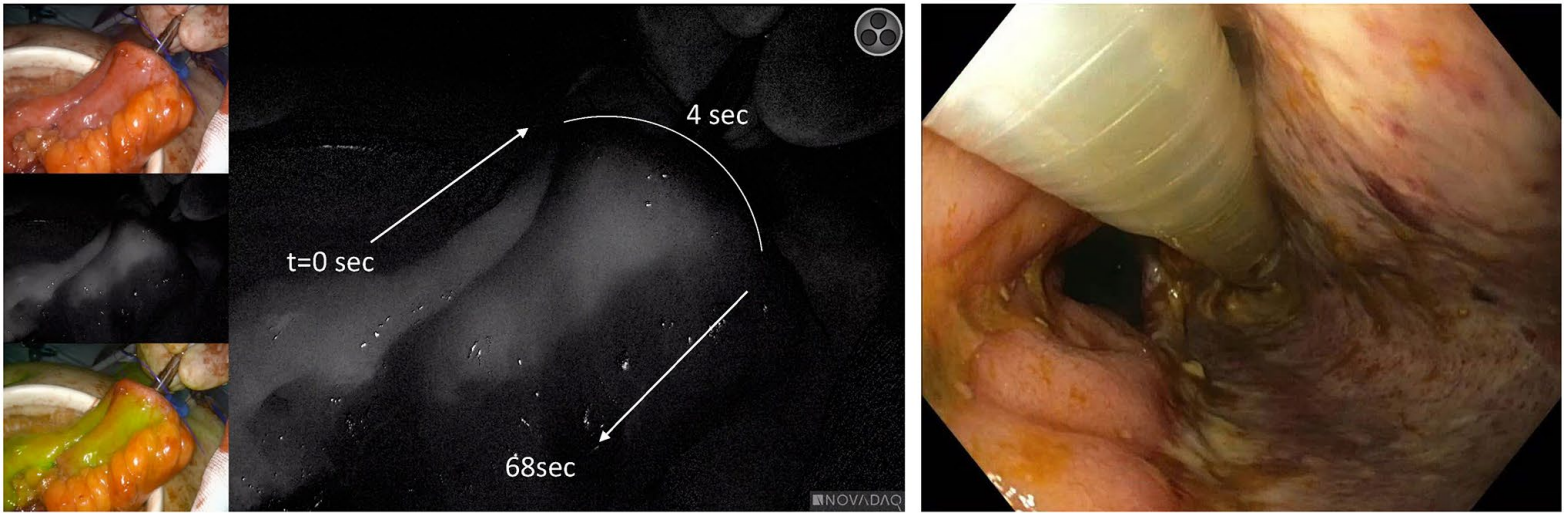
A case with intact but delayed fluorescence and mucosal ischaemia of the blind loop

In 1 case, mucosal ischaemia of the blind loop was observed by endoscopy, after ligation of interconnecting branches without stoma creation (Fig. 3). Time values were 92 s for ICGi-blind loop and 64 s for anvil-blind loop. No anastomotic leakage occurred, the mucosa seemed to re-epithelialize over time and the pouch was preserved. In the case of intact but delayed ICG fluorescence between anvil and blind loop (33 s) after ligation of interconnecting branches leading to primary ileostomy, no anastomotic leakage occurred.

## Discussion

In this study, FA-guided IPAA was described and FA characteristics were evaluated in ileoanal pouches with intact vascularisation as opposed to pouches where lengthening measures required vascular ligation. For the quantitative FA parameter time to fluorescent enhancement, time values were prolonged in patients with vascular ligation, without reaching statistical significance.

FA is of potential added clinical value during IPAA, especially after vascular ligation [8]. Vascular ligation might cause inflow (arterial) or outflow (venous) problems. FA displays problems in arterial flow as absence of ICG in the pouch [8]. Time values were assessed for small bowel parts that were perfused by ICG, translating into intact arterial flow. No significant differences were noticed for time values between patients with or without vascular ligation, although a trend towards prolonged time from ICG injection to first signal was observed. Time values might therefore indicate redirection of arterial flow through the arcade, or presence of venous outflow obstruction. Increased resistance in the venous network might explain the delayed flow in case of venous outflow obstruction. Therefore, FA during IPAA might enable differentiation between an inflow problem (no ICG fluorescence), an outflow problem (delayed, but intact ICG flow) and adequate perfusion (rapid ICG flow). When evaluated in a larger group of patients, one might be able to correlate the degree of delayed flow with patient outcomes, including anastomotic leakage and ischaemia. However, this correlation might be less profound for the pouch as compared to conduits that have a single artery supplying tissue perfusion, e.g. the gastric conduit after esophagectomy or the descending colon after low anterior resection [15, 16]. Small bowel showing intact, but delayed ICG fluorescence might still be used for IPAA, as additional resection of this part might further compromise the balance between perfusion and lengthening. However, these cases may benefit from a 3-stage instead of a modified 2-stage procedure [12].

Absence of an ICG signal in parts of the pouch in 6 cases resulted in a change of management in this study to ascertain IPAA reconstruction with ICG-perfused small bowel. Change in management included additional resection or selecting a more proximal small bowel loop for all types of vascular ligation. Ligation of both the ileocolic trunk as well as interconnecting branches should probably be avoided, especially when no arcade is present and the bowel is vascularised by segmental branches. In this study, this resulted in creation of a new pouch and resection of 25 cm of non-vital terminal ileum. Anastomotic leakage within the vascular ligation group was only observed after ligation of interconnecting branches, in one combined with ileocolic arcade ligation, suggesting more severe perfusion problems after ligation of interconnecting branches. However, anastomotic leakage might also occur because of traction caused by insufficient reach after optimal mesenteric lengthening.

Anastomotic leakage secondary to perfusion restriction might be prevented by optimisation of perfusion under guidance of FA. In a case-matched study of 64 patients [8], only one leak occurred when FA was not applied. An anastomotic leakage rate of 15.8% as found in the present study is similar to that in a historic cohort without use of FA that also included patients from our centre (± 17%) [1]. Interestingly, vascular ligation was more frequently performed when FA was applied (47%) than in a historic control cohort (16%) [8]. In the present cohort, vascular ligation was also regularly performed (39.5%). This might be explained by change in surgical attitude, i. e. becoming more aggressive towards vessel ligation and not accepting minor traction on the anastomosis. However, FA could also provide a sense of security when vascular ligation is performed because perfusion can be checked more objectively. Especially after vessel ligation, it is important to check perfusion by FA as this led to significantly more cases of change in management in this study. Although not routinely done in this cohort, perfusion should ideally be checked using FA before creation of the pouch.

In this study, one fluorescence imaging system with standard settings was used with a standard ICG dose. It is unknown how these findings relate to a setting with a different imaging system and ICG dose. In future studies, calibration of imaging systems is mandatory to identify differences in fluorescence read-out. Furthermore, the fluorescent signal was still interpreted subjectively. To asses separate (inflow) fluorescence parameters of FA more objectively, software that is able to produce fluorescence-time curves is a promising tool for future research (NTR trialregister.nl, NL8653) [10]. An advantage of the curves is that outflow can also be quantified, hopefully enabling to report on (the possible impact of) venous congestion.

Correlation with haemodynamic parameters was evaluated, and only heart rate was significantly correlated. The inverse correlation seems plausible, as time values between ICG injection and arrival at the pouch might prolong when the heart rate decreases. It is important to further study the association between haemodynamics and FA, as FA assessment and its threshold are ideally independent of the patient’s haemodynamic state. Besides mean arterial pressure, heart rate and noradrenaline usage, other potential important parameters to take into account are cardiac output [17], viscosity (haematocrit) and the patient’s temperature. In the future, prediction models created by conventional statistics or by the use of artificial intelligence might take both patient characteristics and FA thresholds into account, which would probably allow for more precise prediction of patient outcomes.

Limitations of this study include small population as no power calculation was performed, retrospective collection of patient and operative data, and a low number of events of vascular ligation and anastomotic leakage. However, this was an explorative study and although patient data were collected retrospectively, vascular ligation was often reported, and when not, it could be checked on surgical recordings. Because of the retrospective nature of this study, patient related outcome measures were not captured. Prospective studies are warranted to include these functional outcomes. Correction for the multifactorial aetiology of anastomotic leakage and stratifying for possible confounders was not possible owing to a low absolute number of events, and this might have led to biased results. Redo pouches were not included in this cohort, however this is an interesting population to verify perfusion and a potential focus for further research to elucidate the role of FA in pouch surgery. However, this is amongst the first studies reporting on FA-guided IPAA, and to our knowledge the first that evaluated a quantitative FA value.

## Conclusions

Results from this study show that FA can differentiate between arterial (no ICG fluorescence) and venous (intact but delayed ICG fluorescence) problems or adequate perfusion (rapid ICG flow). A larger prospective cohort must be conducted to identify potential thresholds for predicting patient outcomes.
